# Hypoxia-Dependent Upregulation of VEGF Relies on β3-Adrenoceptor Signaling in Human Retinal Endothelial and Müller Cells

**DOI:** 10.3390/ijms26094043

**Published:** 2025-04-24

**Authors:** Martina Lucchesi, Lorenza Di Marsico, Lorenzo Guidotti, Matteo Lulli, Luca Filippi, Silvia Marracci, Massimo Dal Monte

**Affiliations:** 1 Department of Biology, University of Pisa, 56126 Pisa, Italy; martina.lucchesi@biologia.unipi.it (M.L.); l.dimarsico@student.unisi.it (L.D.M.); l.guidotti1@student.unisi.it (L.G.); silvia.marracci@unipi.it (S.M.); 2 Department of Experimental and Clinical Biomedical Sciences “Mario Serio”, University of Florence, 50121 Florence, Italy; matteo.lulli@unifi.it; 3 Department of Clinical and Experimental Medicine, Division of Neonatology and NICU, University of Pisa, 56126 Pisa, Italy; luca.filippi@unipi.it

**Keywords:** HIF-1, BARs, NOS enzymes, nitric oxide, MIO-M1 cells, hRECs

## Abstract

β-adrenoceptors (BARs) are involved in vascular endothelial growth factor (VEGF) production during retinal neovascularization. Here, using human retinal endothelial and Müller cells (hRECs and MIO-M1, respectively), we evaluated the effects exerted by hypoxia on BARs, hypoxia-inducible factor-1α subunit (HIF-1α) and VEGF, as well as the involvement of BAR3 and nitric oxide synthase (NOS) enzymes in hypoxia-induced VEGF production. We altered oxygen availability through a hypoxic incubator. BARs, HIF-1 α and VEGF levels were evaluated. Cells were treated with the BAR3 antagonist SR59230A, different NOS inhibitors or the NO donor SNAP. The influence of the BAR3/NOS axis on hypoxic VEGF production was assessed. Hypoxia upregulated BAR3, HIF-1α and VEGF in hRECs and MIO-M1 cells. SR59230A counteracted hypoxia-dependent VEGF increase in both cell lines, exerting no effect on HIF-1α upregulation. Treatments with NOS inhibitors prevented the hypoxia-dependent VEGF increase, while SNAP abrogated the effect of SR59230A in reducing hypoxia-induced VEGF upregulation. The present results corroborate the hypothesis that in the hypoxic retina, BAR3 influence on VEGF production is mediated by NO and suggest that, at least in endothelial and Müller cells, BAR3 activity is necessary to allow the HIF-1-mediated VEGF upregulation.

## 1. Introduction

Through the years, the role played by the G-coupled β-adrenoceptors (BARs) in angiogenesis via modulation of proangiogenic factors has been progressively highlighted [1,2,3,4]. Indeed, stress conditions like hypoxia are known to cause catecholaminergic overstimulation [5], which in turn alters signaling pathways associated with BARs [6]. Pathological angiogenesis, a key event in many hypoxic/ischemic retinal diseases, may rely, at least in part, on increased β-adrenergic transmission [3,6]. Although the role of the β-adrenergic system in vascular endothelial growth factor (VEGF) synthesis appears to be mainly dependent on BAR2 activity through a mechanism involving the hypoxia-inducible factor 1 (HIF-1) [6,7,8], accumulating evidence suggests a possible contribution of BAR3 to angiogenesis. BAR3, the last identified member of the BAR family, was initially shown to regulate lipolysis and thermogenesis [9] and to play important roles in the pathophysiology of the cardiovascular system [10] and the urinary tract [11,12]. In the last decade, BAR3 activity was linked to pathologic angiogenesis in different models of retinal neovascularization [13,14], as well as in murine melanoma [15].

In the oxygen-induced retinopathy (OIR) mouse model of retinopathy of prematurity, BAR3 is the only BAR subtype to be upregulated by hypoxia within the retina [16], being *BAR3* a target gene of HIF-1 [17]. Pioneer experiments investigating the role of BAR3 in the OIR model showed that BAR3 blockade did not affect retinal neovascularization when the model was established in the C57BL/6J strain [7]. On the contrary, when the model was established in the 129S mouse strain, which is characterized by a more pronounced upregulation of BAR3 after hypoxia exposure [18], BAR3 agonism completely abolishes retinal neovascularization while stimulating the physiologic revascularization of the avascular retina [19]. In addition, BAR3 stimulation increases the expression of proangiogenic factors in human retinal and choroidal endothelial cells [20,21] but not in mouse choroidal endothelial cells [22]. Moreover, ex vivo studies in C57BL/6J mouse retinal explants showed that BAR3 activation under hypoxia increases VEGF release, while BAR3 inhibition or silencing drastically downregulates the hypoxic levels of VEGF [13].

A large body of evidence suggests that BAR3 influence on VEGF production is mediated by nitric oxide (NO) signaling pathway, as shown in mouse retinal explants [13], murine melanoma cells [23], human vein endothelial umbilical cells [24], the endothelium of human coronary arteries [25], rat adipocytes [26], the cardiovascular system [27] and the bladder [11]. Indeed, Gi protein activation by BAR3 stimulates the synthesis of NO [28], which is an important modulator of VEGF levels [29,30,31].

NO is generated by three different isoforms of nitric oxide synthase (NOS): neuronal (nNOS), endothelial (eNOS) and inducible (iNOS). nNOS and eNOS are constitutively expressed, calcium-dependent enzymes, that synthesize NO in the nanomolar range, while iNOS is an inducible, calcium-independent enzyme that synthesizes NO in the micromolar range [32]. BAR3-induced NO production was initially associated with eNOS-dependent production of NO in the human ventricle [33]. However, subsequent data indicate that BAR3 can modulate NO expression also via nNOS and iNOS, in the heart and vasculature [34,35]. Particularly, iNOS expression seems to be positively affected by BAR3 activity in mouse hearts [35], rat adipocytes [36] and mouse melanoma cells [23] but not in mouse brown adipose tissue [37]. Interestingly, in mouse retinal explants, BAR3 modulation of VEGF release under hypoxia relies on NOS activity [13].

The present study explores in vitro the putative impact of hypoxia on the modulation of BAR expression using two different human retinal cell lines: retinal endothelial cells (hRECs) and Müller glial cells (MIO-M1). We chose these two models since, within the retina, Müller glia is the main source of VEGF under hypoxic conditions [38,39,40,41], while endothelial cells represent the main target of VEGF, being also able to produce it [2,42]. We also analyzed the expression of HIF-1 and VEGF under hypoxia to investigate their supposed correlation with BAR levels. Using the BAR3 antagonist SR59230A, we then examined whether BAR3 blockade could influence VEGF expression and whether this alteration correlates with changes in NOS expression. Finally, through pharmacologic modulation of NOS activity, we explored the role of NOS enzymes in mediating the effect of BAR3 blockade on VEGF production.

## 2. Results

### 2.1. Impact of Hypoxic Treatment on Cell Proliferation and Viability

**Figure 1:**
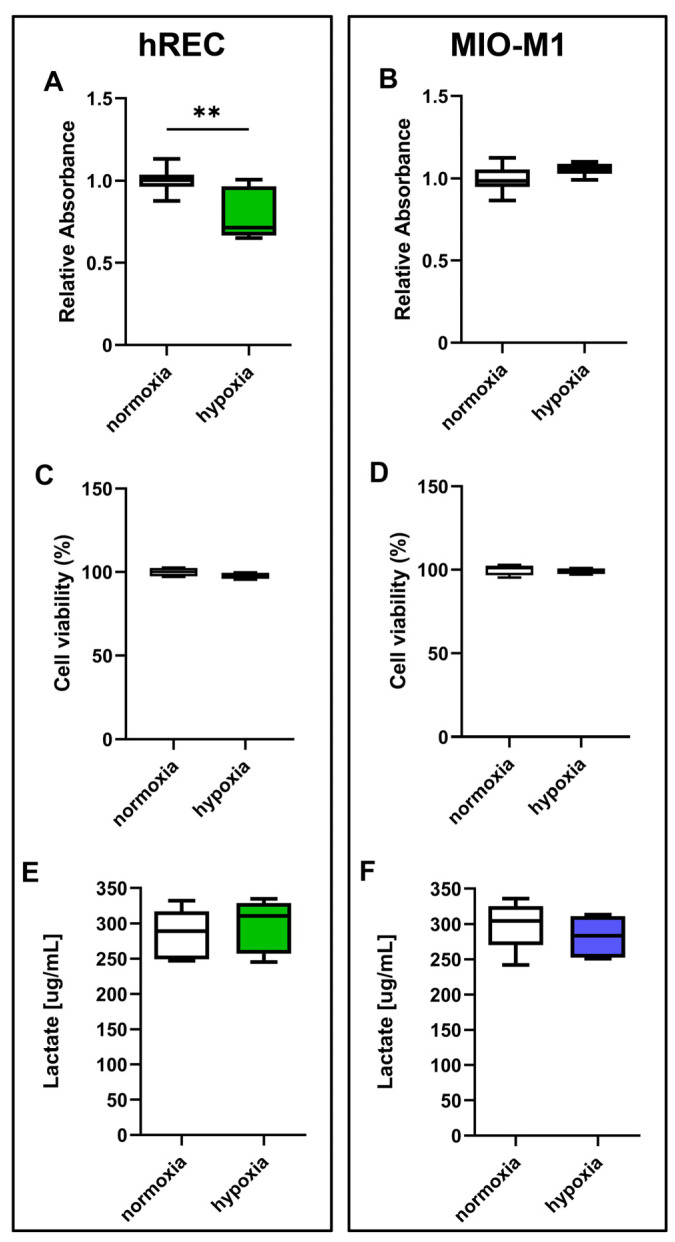
Effect of 24 h hypoxia on human retinal endothelial cell (hREC) and human Müller (MIO-M1) cell proliferation and viability. In both hRECs (**A**,**C**,**E**) and MIO-M1 cells (**B**,**D**,**F**), cell proliferation was measured using the MTT assay (**A**,**B**), while cell viability was evaluated by the trypan blue (**C**,**D**) and lactate concentration measurement (**E**,**F**) assays. Data are shown as box plots with minimum to maximum whiskers and represent the absorbance values normalized to those measured in normoxic controls (*n* = 10 in **A**,**B**, *n* = 5 in **C**–**F**). Statistical significance was evaluated through unpaired *t*-test. ** *p* < 0.01.

Firstly, we examined the impact of hypoxic treatment on cell proliferation. The results obtained through the MTT assay showed that after 24 h hypoxia hREC proliferation was significantly decreased compared to normoxic control (Figure 1A). On the other hand, 24 h hypoxia does not affect MIO-M1 cell proliferation (Figure 1B). However, hypoxia did not affect cell viability, in both hRECs and MIO-M1 cells, as evidenced by the trypan blue (Figure 1C,D) and lactate concentration measurement (Figure 1E,F) assays.

### 2.2. Effect of Hypoxic Treatment on BAR Expression

**Figure 2:**
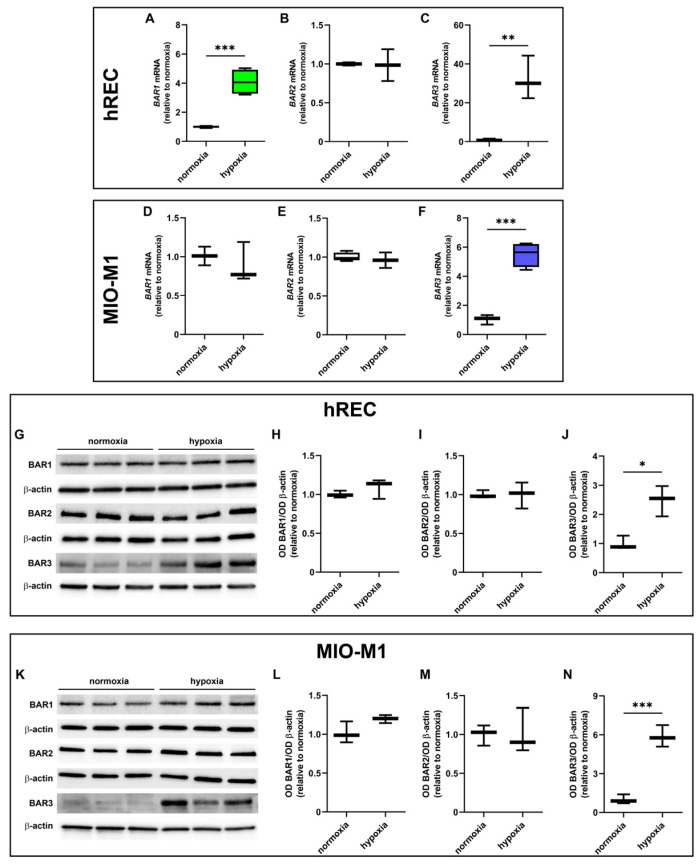
Effect of 24 h hypoxia on β-adrenoceptor (BAR) expression. (**A**–**F**) mRNA levels of BAR1 (**A**,**D**), BAR2 (**B**,**E**) and BAR3 (**C**,**F**) in hRECs (**A**–**C**) and MIO-M1 cells (**D**–**F**). (**G**–**N**) Representative Western blots (**G**,**K**) and densitometric analysis (**H**–**J**,**L**–**N**) of BAR1 (**H**,**L**), BAR2 (**I**,**M**) and BAR3 (**J**,**N**) levels in hRECs (**G**–**J**) and MIO-M1 cells (**K**–**N**). In (**A**–**F**), data were analyzed by the formula 2^−ΔΔCT^ using β-actin as the internal standard and normalized to those measured in normoxic controls (*n* = 3). In (**H**–**J**) and (**L**–**N**), β-actin was used as the loading control, and optical density (OD) values were normalized to those measured in normoxic controls (*n* = 3). Data are shown as box plots with minimum to maximum whiskers. Statistical significance was evaluated through unpaired *t*-test. * *p* < 0.05, ** *p* < 0.01 and *** *p* < 0.001.

The expression levels of BAR1, BAR2 and BAR3 were evaluated in cells exposed to hypoxia. At the transcriptome level, 24 h hypoxic treatment increased BAR1 mRNA in hRECs and BAR3 mRNA in both cell lines, while it did not affect mRNA expression of either BAR1 in MIO-M1 cells or BAR2 in both cell lines (Figure 2A–F). At the protein level, 24 h hypoxia did not influence BAR1 and BAR2 while it significantly increased BAR3 protein expression in both cell lines (Figure 2G–L).

### 2.3. Effect of Hypoxic Treatment on HIF-1α and VEGF Levels

**Figure 3:**
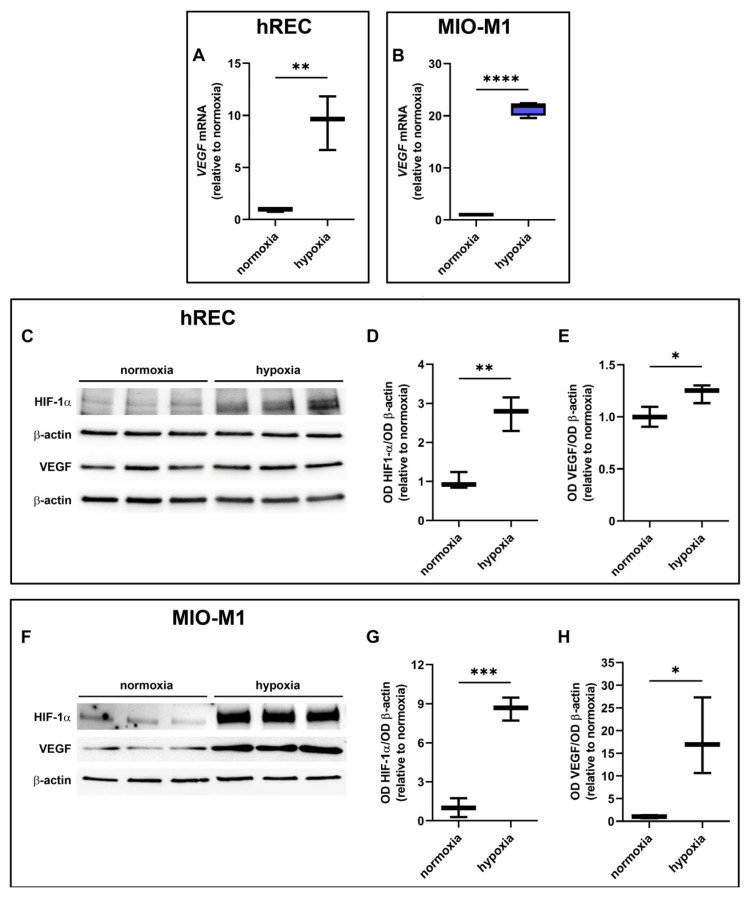
Effect of 24 h hypoxia on hypoxia-inducible factor (HIF)-1α and vascular endothelial growth factor (VEGF) expression. (**A**,**B**) mRNA levels of *VEGF* in hRECs (**A**) and MIO-M1 cells (**B**). (**C**–**H**) Representative Western blots (**C**,**F**) and densitometric analysis (**D**,**E**,**G**,**H**) of HIF-1α (**D**,**G**) and VEGF (**E**,**H**) levels in hRECs (**C**–**E**) and MIO-M1 cells (**F**–**H**). In (**A**,**B**), data were analyzed by the formula 2^−ΔΔCT^ using β-actin as the internal standard and normalized to those measured in normoxic controls (*n* = 3). In (**D**,**E**,**G**,**H**), β-actin was used as the loading control and OD values were normalized to those measured in normoxic controls (*n* = 3). Data are shown as box plots with minimum to maximum whiskers. Statistical significance was evaluated through unpaired *t*-test. * *p* < 0.05, ** *p* < 0.01, *** *p* < 0.001 and **** *p* > 0.0001.

Next, we analyzed the effects of hypoxia on HIF-1α and VEGF expression. A significant upregulation of VEGF mRNA was observed in both cell lines following 24 h hypoxic treatment (Figure 3A,B). In addition, HIF-1α and VEGF protein were strongly increased in both cell lines after 24 h hypoxia (Figure 3C–H).

### 2.4. Effect of SR59230A on Cell Proliferation and Viability

**Figure 4:**
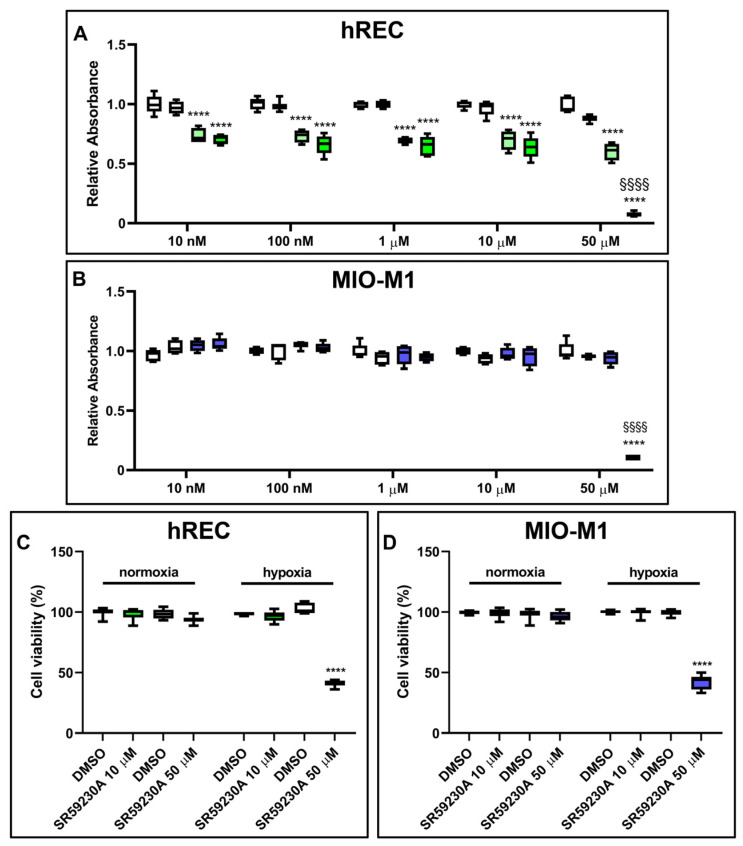
Effect of 24 h treatment with SR59230A on hRECs and MIO-M1 cell proliferation and viability in normoxia and hypoxia. Cell proliferation of HRECs (**A**) and MIO-M1 (**B**) was analyzed through the MTT assay, while cell viability in cells exposed to 50 µM SR59230A was measured by the trypan blue assay (**C**,**D**). Cells were cultured in normoxia (first and second boxes in each data set in **A**,**B**; first data set in **C**,**D**) or hypoxia (third and fourth boxes in each data set in **A**,**B**; second data set in **C**,**D**) in the presence of the vehicle (dimethyl sulfoxide, DMSO; first and third boxes in each data set in **A**,**B**; as indicated in **C**,**D**) or the reported concentrations of SR59230A (second and fourth boxes in each data set in **A**,**B**; first data set in **C**,**D**; as indicated in **C**,**D**). Data are shown as box plots with minimum to maximum whiskers and represent the absorbance values normalized to those measured in DMSO-treated normoxic controls (*n* = 5 in **A**,**B**, *n* = 8 in **C**,**D**). Statistical significance was evaluated through two-way ANOVA followed by Tukey’s multiple comparisons post hoc test. In **A**,**B**, **** *p* < 0.0001 versus the corresponding DMSO-treated or SR59230A-treated normoxic cells; ^§§§§^ *p* < 0.0001 versus the corresponding DMSO-treated hypoxic cells. In **C**,**D**, **** *p* < 0.0001 versus the corresponding DMSO-treated hypoxic cells.

We assessed whether SR59230A had any impact on hREC and MIO-M1 cell proliferation in either normoxic or hypoxic conditions. Particularly, we analyzed cell proliferation in response to SR59230A ranging from 10 nM to 50 µM. The vehicle did not alter hREC and MIO-M1 cell proliferation in normoxia and hypoxia compared to untreated cells (Supplementary Figure S1). In normoxia, SR59230A did not alter either hREC or MIO-M1 cell proliferation. In hypoxia, a condition that per se reduced hREC but not MIO-M1 cell proliferation, SR59230A heavily affected cell proliferation only at 50 µM (Figure 4A,B). Cell viability, as evaluated by the trypan blue assay, was not affected by SR59230A in normoxia, while in hypoxia, SR59230A heavily reduced cell viability in both cell lines at 50 µM but not at 10 µM (Figure 4C,D).

Although SR59230A at 10 µM did not alter the proliferation of hRECs, it reduced their migration, as evaluated in a wound healing assay (Supplementary Materials and Methods, Supplementary Results and Supplementary Figure S2). Based on these results, to avoid the toxic effects of the drug on hypoxic cells, we decided to use SR59230A at 10 µM for the subsequent experiments.

### 2.5. Effect of SR59230A on HIF-1α and VEGF Levels

**Figure 5:**
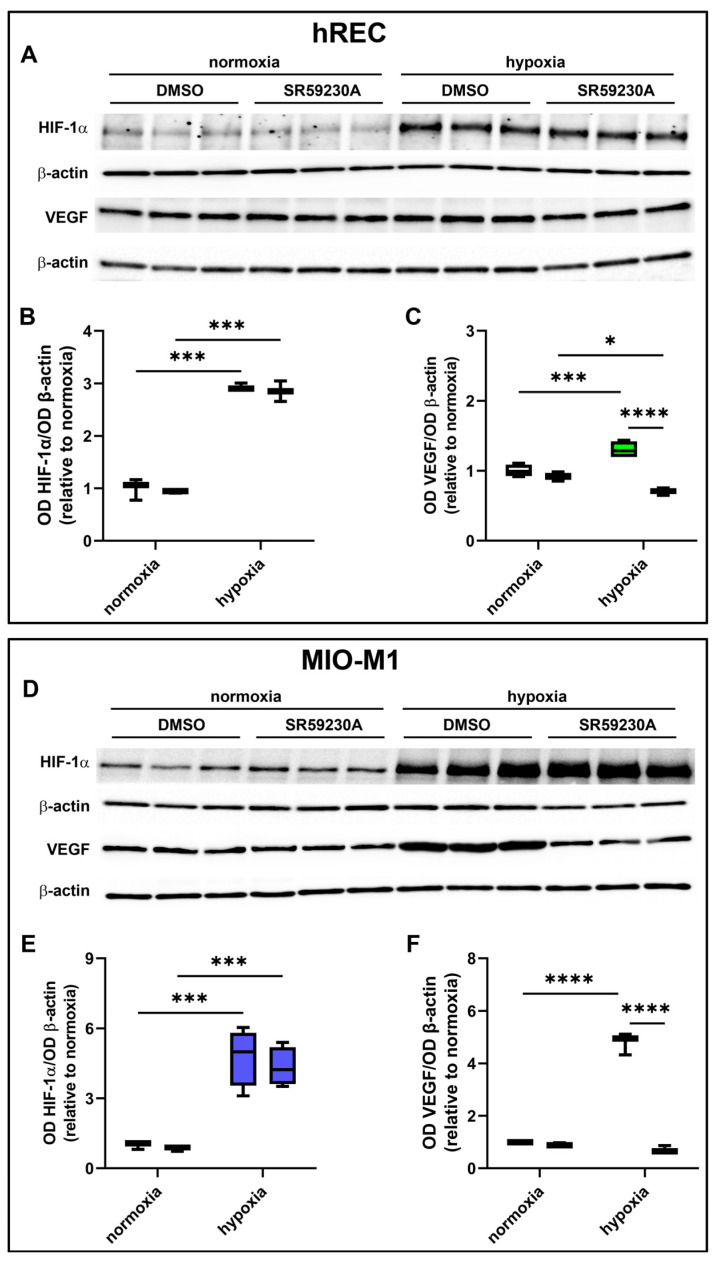
Effect of 10 µM SR59230A on HIF-1α and VEGF expression in cells exposed to normoxia or 24 h hypoxia. (**A**–**F**) Representative Western blots (**A**,**D**) and densitometric analysis (**B**,**C**,**E,F**) of HIF-1α (**A**,**B**,**D**,**E**) and VEGF (**A**,**C**,**D**,**F**) levels in hRECs (**A**–**C**) and MIO-M1 cells (**D**–**F**). Cells were cultured in the presence of the vehicle (DMSO; first box in each data set) or SR59230A (second box in each data set). β-actin was used as the loading control, and OD values were normalized to those measured in DMSO-treated normoxic controls (*n* = 3). Data are shown as box plots with minimum to maximum whiskers. Statistical significance was evaluated through two-way ANOVA followed by Tukey’s multiple comparisons post hoc test. * *p* < 0.05, *** *p* < 0.001 and **** *p* < 0.0001.

hRECs and MIO-M1 cells were treated with SR59230A to investigate whether BAR3 plays a role in regulating VEGF production in response to hypoxia and whether HIF-1α plays a role in this. As shown by the present results, SR59230A did not affect HIF-1α levels either in hRECs (Figure 5A,B) or in MIO-M1 cells (Figure 5D,E). Indeed, no significant differences in HIF-1α expression were observed between vehicle- and SR59230A-treated cells, both in normoxia and in hypoxia. On the other hand, the hypoxia-dependent VEGF increase was prevented by SR59230A treatment in both cell lines (Figure 5A,C,D,F). Notably, VEGF levels in SR59230A-treated hypoxic hRECs were significantly lower than in normoxia.

### 2.6. Effect of SR59230A on NOS Expression

**Figure 6:**
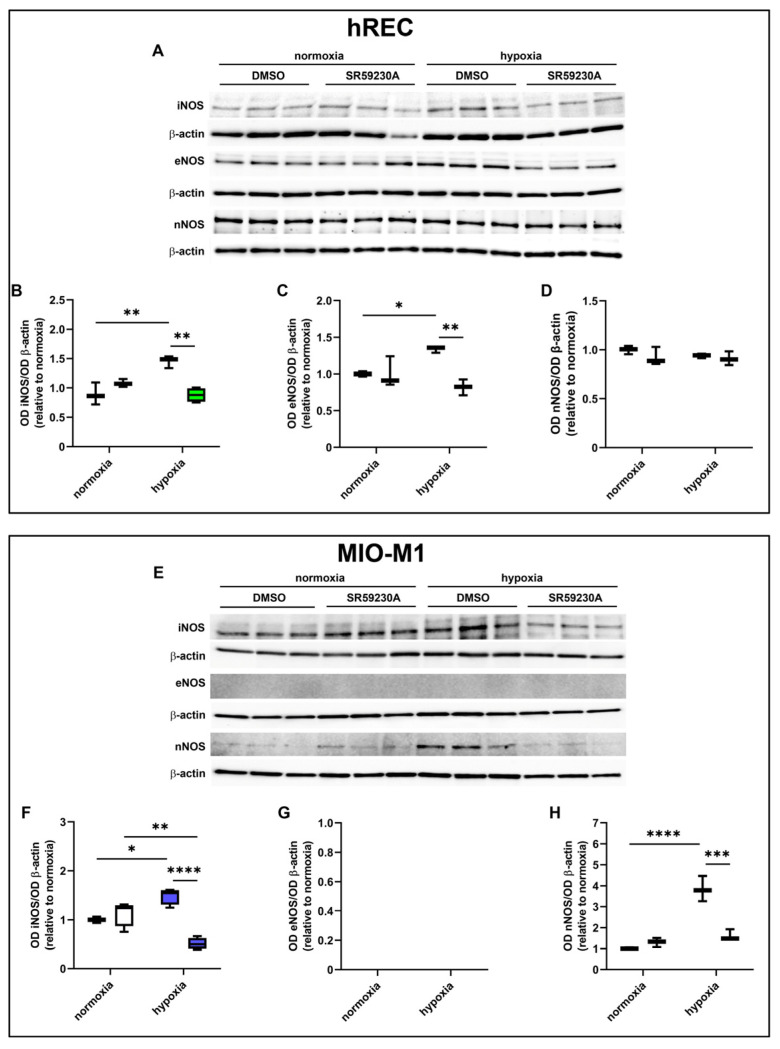
Effect of 10 µM SR59230A on the expression of nitric oxide synthase (NOS) enzymes in cells exposed to normoxia or 24 h hypoxia. (**A**–**H**) Representative Western blots (**A**,**E**) and densitometric analysis (**B**–**D**,**F**–**H**) of iNOS (**B**,**F**), eNOS (**C**,**G**) and nNOS (**D**,**H**) levels in hRECs (**A**–**D**) and MIO-M1 cells (**E**–**H**). Cells were cultured in the presence of the vehicle (DMSO; first box in each data set) or SR59230A (second box in each data set). β-actin was used as the loading control, and OD values were normalized to those measured in DMSO-treated normoxic controls (*n* = 3). Data are shown as box plots with minimum to maximum whiskers. Statistical significance was evaluated through two-way ANOVA followed by Tukey’s multiple comparisons post hoc test. * *p* < 0.05, ** *p* < 0.01, *** *p* < 0.001 and **** *p* < 0.0001.

We then sought possible molecular mechanisms involved in the VEGF decrease observed in SR59230A-treated hypoxic cells. Particularly, we investigated whether the expression of the different NOS isoforms (iNOS, eNOS, nNOS) was affected by drug treatment (Figure 6). Twenty-four hour hypoxia significantly increased iNOS expression in both cell lines, in which SR59230A prevented the hypoxia-induced iNOS upregulation (Figure 6A,B,E,F). In hRECs, eNOS was upregulated by hypoxia, an effect that was prevented by SR59230A (Figure 6A,C), while in MIO-M1 cells, no expression of eNOS could be observed (Figure 6E,G). In hRECs, neither hypoxia nor SR59230A altered the expression level of nNOS (Figure 6D). On the other hand, in MIO-M1 cells, nNOS was remarkably upregulated by hypoxia, and SR59230A brought its expression level to that of normoxic controls (Figure 6H).

### 2.7. Effect of NOS Antagonism on VEGF Levels

**Figure 7:**
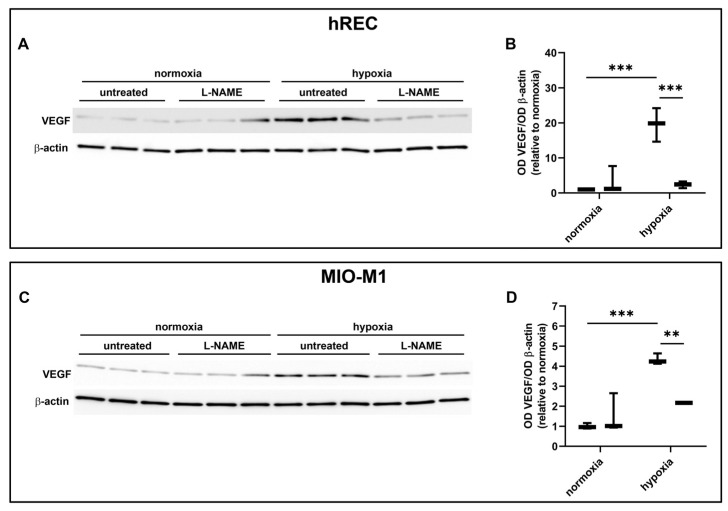
Effect of the panNOS inhibitor L-NAME at 5 mM on the expression of VEGF in cells exposed to normoxia or 24 h hypoxia. (**A**–**D**) Representative Western blots (**A**,**C**) and densitometric analysis (**B**,**D**) of VEGF levels in hRECs (**A**,**B**) and MIO-M1 cells (**C**,**D**). Cells were cultured in the absence (untreated; first box in each data set) or in the presence of L-NAME (second box in each data set). β-actin was used as the loading control, and OD values were normalized to those measured in untreated normoxic controls (*n* = 3). Data are shown as box plots with minimum to maximum whiskers. Statistical significance was evaluated through two-way ANOVA followed by Tukey’s multiple comparisons post hoc test. ** *p* < 0.01 and *** *p* < 0.001.

**Figure 8:**
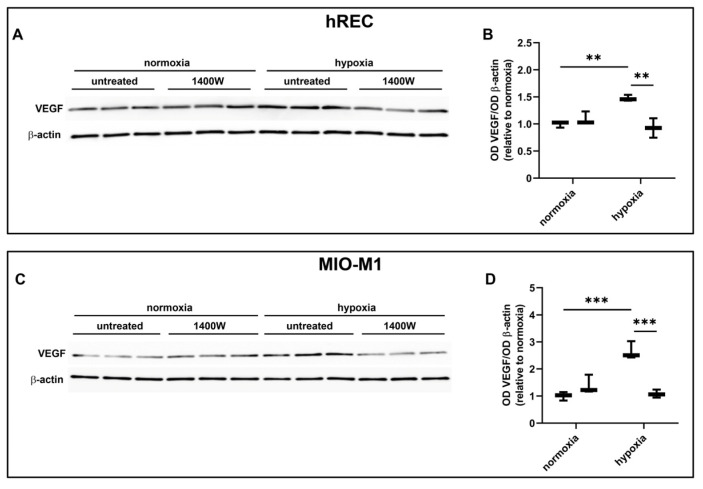
Effect of the inducible NOS (iNOS) inhibitor 1400W at 50 µM on the expression of VEGF in cells exposed to normoxia or 24 h hypoxia. (**A**–**D**) Representative Western blots (**A**,**C**) and densitometric analysis (**B**,**D**) of VEGF levels in hRECs (**A**,**B**) and MIO-M1 cells (**C**,**D**). Cells were cultured in the absence (untreated; first box in each data set) or in the presence of 1400W (second box in each data set). β-actin was used as the loading control, and OD values were normalized to those measured in untreated normoxic controls (*n* = 3). Data are shown as box plots with minimum to maximum whiskers. Statistical significance was evaluated through two-way ANOVA followed by Tukey’s multiple comparisons post hoc test. ** *p* < 0.01 and *** *p* < 0.001.

In these experiments, we evaluated the contribution of the NOS isoforms on hypoxia-induced VEGF production in both hRECs and MIO-M1 cells. Particularly, we used the panNOS inhibitor L-NAME and the selective iNOS inhibitor 1400W. L-NAME at 5 mM prevented the hypoxia-dependent VEGF protein upregulation in both cell lines (Figure 7), an effect that was mimicked by 1400W at 50 µM (Figure 8).

### 2.8. Effect of NOS Activation and SR59230A on VEGF Levels

**Figure 9:**
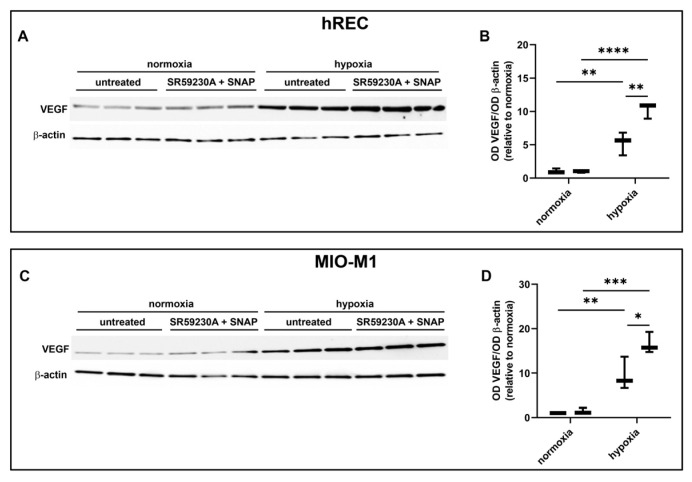
Effect of 10 µM SR59230A in the presence of the nitric oxide donor SNAP at 3 µM on the expression of VEGF in cells exposed to normoxia or 24 h hypoxia. (**A**–**D**) Representative Western blots (**A**,**C**) and densitometric analysis (**B**,**D**) of VEGF levels in hRECs (**A**,**B**) and MIO-M1 cells (**C**,**D**). Cells were cultured in the absence (untreated; first box in each data set) or in the presence of SR59230A and SNAP (second box in each data set). β-actin was used as the loading control, and OD values were normalized to those measured in untreated normoxic controls (*n* = 3). Data are shown as box plots with minimum to maximum whiskers. Statistical significance was evaluated through two-way ANOVA followed by Tukey’s multiple comparisons post hoc test. * *p* < 0.05, ** *p* < 0.01, *** *p* < 0.001 and **** *p* < 0.0001.

Finally, we evaluated in both hRECs and MIO-M1 the effects of NOS stimulation on VEGF production using the NO donor SNAP in the presence of 10 µM SR59230A. In both cell lines, SNAP at 3 µM blocked the inhibitory effect of SR59230A on hypoxia-induced upregulation of VEGF production, which was further increased with respect to untreated hypoxic cells (Figure 9).

## 3. Discussion

Within the hypoxic retina, (i) Müller cells are the main producers of VEGF [38,39,40,41], (ii) endothelial cells represent the main receivers of VEGF [2,42], and (iii) both these two cell types express all the three BAR isoforms, as shown by the present results. Therefore, MIO-M1 cells and hRECs are good in vitro models to study the possible correlation between BAR expression and VEGF production in hypoxia.

First of all, we investigated whether hypoxia impacts cell proliferation and viability. As shown here, hypoxia has no effects on MIO-M1 cells. On the other hand, hREC proliferation, but not viability, is affected by 24 h hypoxia, as previously observed in endothelial cells exposed to reduced oxygen tension [43,44,45]. In particular, in hRECs, 24 h hypoxia seems to induce a reduction in mitochondrial function but not cytotoxicity [44], in line with our findings.

Then, we evaluated whether BAR expression was affected by oxygen deprivation. BAR1 level is not strongly affected by hypoxia in the two cell lines, with an increase in *BAR1* mRNA observed only in hRECs. However, previous in vivo studies in OIR mice tend to exclude a role for BAR1 in VEGF modulation in the retina [7]. Moreover, BAR1 agonism was ineffective in inducing hREC proliferation and migration [20], two processes mediated by VEGF signaling [46]. This suggests that BAR1 contribution to pathological angiogenesis and VEGF production is unlikely.

BAR2 expression was not affected at all by the hypoxic treatment in the two cell lines, in agreement with previous studies in OIR mice [16]. However, BAR2 blockade decreases VEGF retinal levels and reduces pathogenic neovascularization in OIR mice [7]. Similar effects were obtained through BAR2 desensitization, due to prolonged stimulation with the BAR2 agonist isoproterenol [6]. Furthermore, the knock-out of BAR1/2 in OIR mice prevents the formation of the avascular area within the retina and provides protection against neovascularization [47]. These data suggest that BAR2 could be involved in pathological angiogenesis, even though its expression is not altered by hypoxia.

BAR3 was the most responsive BAR isoform to hypoxia, at least in the two cell lines used in this study. Previous studies highlighted the hypoxia-mediated upregulation of BAR3 in tumor and healthy tissues, including the retina [13,15,16,17,18,48,49,50,51]. However, these are the first results showing a significant hypoxia-dependent BAR3 upregulation in human retinal cells. The canonical-hypoxia response element localized at position -3546 in the human *ADRB3* gene [52] could be responsible for this upregulation at the transcriptional level. In line with this, it was shown that under hypoxia, HIF-1 modulates the mouse *ADRB3* gene via binding to an HIF-binding site that is also present in the corresponding region of the human *ADRB3* gene [17], suggesting that also in human *BAR3* mRNA production could be regulated by hypoxia in an HIF-1-dependent way.

As shown here, SR59230A can efficiently prevent VEGF protein overexpression in both hypoxic hRECs and MIO-M1 cells, suggesting that BAR3 signaling can influence VEGF production in hypoxic conditions, as previously shown in hypoxic mouse retinal explants [13]. On the other hand, it was previously shown in OIR mice that systemic treatment with SR59230A does not affect hypoxic VEGF levels while modulating PKA activity [7], suggesting that the retina needs to be provided with a sufficient amount of SR59230A in order to interfere with the hypoxic production of VEGF. In addition, in the OIR model, hypoxia corresponds to the atmospheric oxygen pressure while retinal cells (current work) and retinal explants [13] are exposed to 1% oxygen, suggesting that this difference between in vivo and in vitro/ex vivo models could contribute to the discrepant results obtained with the different experimental models. Notably, in the OIR model set up in the 129S mouse strain, which responds to hypoxia with a dramatic increase in BAR3 expression [17,18], hypoxia-induced neovascularization and VEGF upregulation may be prevented by BAR3 agonism that stimulates physiologic revascularization of the avascular area [19], suggesting that when particularly overexpressed BAR3 may associate with a regular development of retinal vessels. This indicates that BAR3 density may have a central role in determining the response of retinal BAR3 to BAR3-targeting drugs, and that depending on the context BAR3 blockade [13,15,23,48,50] or BAR3 activation [19] may prevent the development of pathological conditions.

HIF-1 is an important stimulator of *VEGF* transcription, although additional transcription factors and molecules may participate in inducing *VEGF* gene expression under both physiological and pathological conditions [53,54]. As shown by the present results, the decrease in hypoxic VEGF levels following SR59230A treatment occurs in the presence of a persistent upregulation of HIF-1α, suggesting that BAR3 blockade acts on VEGF levels through a pathway that does not involve HIF-1. Among alternative pathways acting upstream VEGF, we considered the NO pathway, as its modulation of VEGF production in different biological systems, including the retina, is widely reported [13,55,56,57].

Among NOS isoforms, iNOS has been identified as an HIF-1 target gene in human cells [53]. As shown here, in hRECs and MIO-M1 cells, hypoxia significantly increases iNOS expression, in line with previous findings in rat retinas exposed to hypoxia and in Müller cells from patients suffering from diabetic retinopathy [58,59]. Of note, in murine cells, hypoxia-induced HIF-1 activity is sufficient to promote iNOS expression [60,61], while in human cells, hypoxia-induced HIF-1 activity is necessary but not sufficient to increase iNOS expression, requiring additional factors [56].

As shown by the present results, eNOS is not expressed in MIO-M1 cells, in line with previous results in mouse Müller cells [62]. On the contrary, eNOS is a marker of endothelial cells, where it is involved in the regulation of vascular function [63,64], in the maintenance of endothelial homeostasis, in both health and disease [65], and is the main NOS isoform involved in NO production [66]. Regarding the effects of hypoxia on eNOS levels, contradictory results are reported, likely because different levels of hypoxia and times of exposure to hypoxia may result in increased or decreased eNOS expression in different cell types [67,68,69,70,71]. As shown here, 24 h hypoxia increases the expression of eNOS in hRECs, in agreement with previous findings indicating eNOS as an HIF-1 target gene in endothelial cells of both humans and rodents [72,73].

Different studies have shown that nNOS is present in the vascular endothelium and contributes to the homeostasis of the cardiovascular system [74,75]. Accordingly, hRECs show nNOS expression, which is insensitive to hypoxia, as shown by the present results. On the other hand, MIO-M1 cells express nNOS, in agreement with previous findings [76], and this expression is significantly increased by hypoxia, in line with previous evidence in human astrocytes [77].

Overall, here, we demonstrate that, despite HIF-1α stabilization not being affected, SR59230A is effective in reducing upregulated NOS levels, suggesting a dual signaling mechanism in which, together with HIF-1α stabilization, BAR3 activity is necessary to sustain NOS expression in hRECs and MIO-M1 cells subjected to hypoxia.

NOS activity seems to be relevant for BAR3-induced VEGF production in hypoxic hRECs and MIO-M1 cells. Indeed, our results show that pharmacological inhibition of all NOS isoforms or specific inhibition of iNOS, previously indicated as the main NOS isoform associated with BAR3 [13,23], decreases VEGF levels, thus mimicking the effects of SR59230A. Moreover, in both hRECs and MIO-M1 cells, the effects of BAR3 blockade are prevented by the NO donor SNAP that, bypassing the reduced expression of NOS enzymes induced by SR59230A, restores the hypoxia-induced increase in VEGF production. These results suggest that NO production via NOS activity belongs to a signaling cascade downstream of BAR3 activation, which is required to induce VEGF production in hypoxic conditions, in line with previous results obtained in the mouse retina [13].

## 4. Materials and Methods

### 4.1. Cell Culture

hRECs constitute a primary human cell line composed of a mixture of venous and arterial cell populations [78]. hRECs were cultured in Endothelial Basal Medium-2 (EBM-2; Lonza, Walkersville, MD, USA), supplemented with 10% Fetal Bovine Serum (FBS) and endothelial growth factors (Microvascular Endothelial Cell Growth Medium-2, EGM-2MV SingleQuot; Lonza). MIO-M1 cells constitute a continuous cell line isolated from a healthy human retina, and they behave like human Müller glia [79]. MIO-M1 cells were cultured in Dulbecco’s modified Eagle’s medium high glucose (4.5 g/L), supplemented with 1% Glutamine, 10% FBS, 1% non-essential amino acids and 1% Penicillin/Streptomycin. hRECs were from a commercial source (Cell Systems, Kirkland, WA, USA), while MIO-M1 cells were provided by Dr. Gloria Astrid Limb (Division of Ocular Biology and Therapeutics, UCL Institute of Ophthalmology, London, UK). Both cell lines were maintained at 37 °C, in 5% CO_2_ in humified conditions. For Western blot experiments and for pharmacological treatment followed by Western blot experiments, cell seeding density was 10^6^ cells per well in TC100 petri dish. For quantitative Real-Time PCR (qRT-PCR) experiments, cell seeding density was 4 × 10^5^ cells per well in a TC60 petri dish.

### 4.2. Hypoxic Treatment

hRECs and MIOM1 cells were starved for 24 h, and then hypoxic conditions were achieved using an incubator (Steri-Cycle i160 CO_2_ incubator, ThermoFisher, Waltham, MA, USA) filled with N2 and 5% CO_2_. The oxygen concentration was constantly monitored and maintained at 1 ± 0.1%, a concentration generally used to mimic severe hypoxia. Cells were incubated in these conditions for 24 h. Normoxic conditions (21% O_2_, 5% CO_2_) were used as a control.

### 4.3. Pharmacologic Treatments

Since its description as a selective BAR3 antagonist [80,81], SR59230A (3-(2-Ethylphenoxy)-1-[[(1S)-1,2,3,4-tetrahydronaphth-1-yl]amino]-(2S)-2-propanol oxalate salt; Merck Life Science, Milano, Italy) has been widely used to inhibit BAR3 activity in vivo, especially in mice [7,48,82,83], and also in vitro [49,50,84,85]. Nevertheless, there is evidence claiming that it might also act as a partial BAR3 agonist [86,87] that it could not suppress BAR3 constitutive activity [88], that it could also bind human BAR1 and BAR2 [89,90], and that it can also inhibit α1-adrenoceptors activity [91,92,93]. SR59230A was dissolved in dimethyl sulfoxide (DMSO) and then diluted in the proper cell culture media, obtaining the final concentrations that were used for the MTT analyses. Cells to be analyzed in Western blot experiments were treated with SR59230A at 10 µM. The corresponding concentrations of DMSO were used as controls.

L-NAME (Nω-nitro-L-arginine methyl ester hydrochloride; Merck Life Science), 1400W (N-[[3-(aminomethyl)phenyl]methyl]-ethanimidamide dihydrochloride; Santa Cruz Biotechnology, Dallas, TX, USA) are a panNOS and an iNOS antagonist, respectively. Particularly, 1400W inhibits iNOS 500-fold more effectively than eNOS and 200-fold more effectively than nNOS [94]. All drugs were dissolved in phosphate-buffered saline (PBS). According to previous data [23], the final concentrations in the culture media of the antagonists used in this work were 5 mM and 50 µM, respectively.

SNAP (S-Nitroso-N-acetyl-DL-penicillamine; Merck Life Science) is a NO donor able to activate NOS isoforms [95]. SNAP was dissolved in PBS, and the final concentration in the culture media used in this work was 3 μM, according to preliminary experiments (Supplementary Figure S3). SNAP was used together with 10 µM SR59230A.

### 4.4. MTT Cell Proliferation Assay

Cells from both lines were seeded onto 96-well flat-bottom microplates. The seeding density was 5.6 × 10^3^ cells per well. Cells were starved for 24 h and then exposed to 24 h hypoxia treatment. Cell proliferation was analyzed through Cell Proliferation Kit I MTT assay (Roche, Monza, Italy). The MTT assay was performed according to the manufacturer’s instructions, and absorbance at 570 nm was measured using a plate reader (Model 680 XR Microplate Reader; Bio-Rad Laboratories, Inc., Hercules, CA, USA).

### 4.5. Trypan Blue Cell Viability Assay

Cells from both lines were seeded onto 96-well flat-bottom plates. The seeding density was 5.6 × 10^3^ cells per well. Cells were starved for 24 h and then exposed to 24 h hypoxia treatment. Cell viability was analyzed through the trypan blue dye exclusion test to measure the number of living and dead cells. After detaching cells from the plate with 0.5% trypsin/EDTA, cell suspensions were added with FBS to inhibit trypsin and centrifuged. The pellets were resuspended in fresh medium, and an aliquot of 10 µL of each suspension was added with 10 µL of 0.4% trypan blue stain (Bio-Rad Laboratories, Inc.) and counted in an automated cell counter (TC20 automated cell counter; Bio-Rad Laboratories, Inc.).

### 4.6. Lactate Concentration Measurement Assay

Cells from both lines were seeded onto 24-well flat-bottom plates. The seeding density was 3 × 10^4^ cells per well. Cells were starved for 24 h and then exposed to 24 h hypoxia treatment. Equal volumes of culture medium were collected to evaluate the amount of L-lactate released as a consequence of cell membrane damage. This colorimetric method used two sequential oxido-reduction reactions, as described previously [96]. In the first reaction, L-lactate was converted into pyruvate and H_2_O_2_ by the stereospecific L-lactate oxidase. In the second reaction, catalyzed by horse radish peroxidase, the chromogenic substrate 2,2′-azino-bis(3-ethylbenzothiazoline-6-sulfonic acid (Merck Life Science) was oxidized by H_2_O_2_ produced in the first reaction and spectrophotometrically measured at 420 nm (FLUOstar Omega; BMG LABTECH, Ortenberg, Germany). L-lactate concentration was calculated from a standard curve of known L-lactate concentrations.

### 4.7. RNA Extraction and qRT-PCR

Total RNA was extracted and purified with a commercially available kit (RNeasy Mini Kit; Qiagen, Hilden, Germany). After the extraction, total RNA was resuspended in RNAse-free water and quantified by spectrophotometry (BioSpectrometer basic; Eppendorf, Hamburg, Germany). Before cDNA synthesis, RNA samples were also tested for the absence of genomic DNA. cDNA was synthesized starting from 1 µg of total RNA using a commercially available kit (QuantiTect^®^ Reverse Transcription Kit; Qiagen). qRT-PCR analysis was performed using a master mix (SsoAdvanced Universal SYBR Green Supermix; Bio-Rad Laboratories, Inc.) on a detection system (CFX Connect Real-Time PCR Detection System provided with the software CFX manager version 3.1; Bio-Rad Laboratories, Inc.). Primer sets were designed to hybridize unique regions of the appropriate gene sequence according to published cDNA sequences in the GenBank database (Table 1). The expression levels were quantified using the comparative 2^−ΔΔCt^ method [97] and were normalized to *β-actin* expression level (used as an endogenous control).

### 4.8. Protein Extraction and Western Blot

Protein extraction was performed using RIPA lysis buffer (Santa Cruz Biotechnology) supplemented with protease inhibitor cocktail, sodium fluoride, sodium orthovanadate and phenylmethanesulfonyl fluoride (Santa Cruz Biotechnology). Protein concentration was determined using a commercially available kit (Micro BCA™ Protein Assay Kit; ThermoFisher). Aliquots of each sample containing equal amounts of protein (15 μg) were subjected to sodium dodecyl sulfate–polyacrylamide gel (4–20%; Bio-Rad Laboratories, Inc.) electrophoresis and then transferred onto nitrocellulose membranes (Bio-Rad Laboratories, Inc.). Blots were blocked in 5% skimmed milk for 1 h at room temperature and then incubated overnight at 4 °C with the specific primary antibodies. After being rinsed, blots were finally incubated for 1 h at room temperature with appropriate peroxidase-labeled secondary antibodies. Codes and dilutions of primary and secondary antibodies are reported in Table 2. Among these antibodies, those targeting BARs have been validated using MIO-M1 cells silenced using specific siRNAs, since the selectivity of commercially available antibodies against BARs may present some concerns (Supplementary Materials and Methods, Supplementary Results and Supplementary Figure S4) [98]. The signal was revealed by chemiluminescence (Clarity Western enhanced chemiluminescence substrate or Clarity Max Western enhanced chemiluminescence substrate; Bio-Rad Laboratories, Inc.). Images were acquired (ChemiDoc XRS+ instrument; Bio-Rad Laboratories, Inc.), and the optical density (OD) of the bands was evaluated (Image Lab 6.0.1 software; Bio-Rad Laboratories, Inc.). Data were normalized to the relative OD of β-actin used as a loading control.

### 4.9. Statistics

Statistical analysis was performed using unpaired Student *t*-test, one-way ANOVA followed by post hoc Tukey’s multiple comparisons or two-way ANOVA followed by post hoc Tukey’s multiple comparisons test, as appropriate. Statistical significance was determined (Prism 8.0.2 software; GraphPad, Boston, MA, USA). Differences with *p* < 0.05 were considered statistically significant. Data are expressed as box-and-whisker plots, with boxes representing the 25th to 75th percentiles and whiskers indicating the minimum and maximum values.

## 5. Conclusions

**Figure 10:**
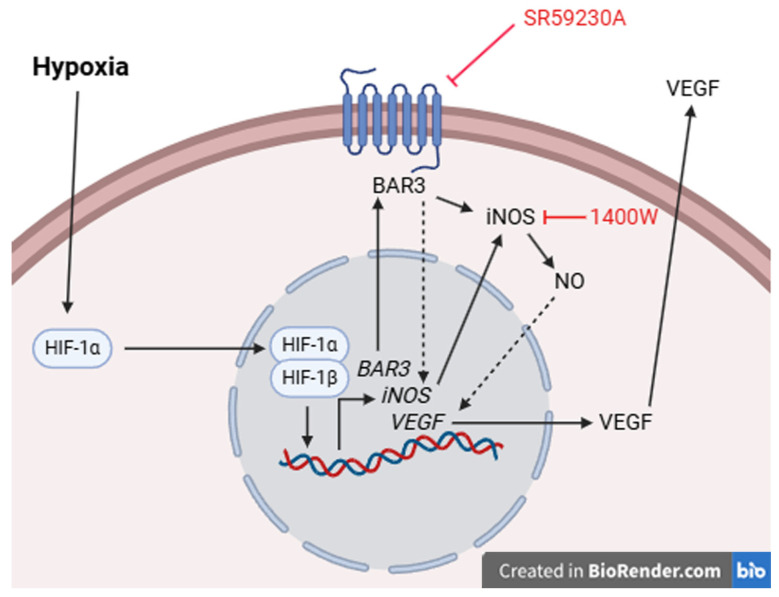
Schematic diagram depicting a possible mechanism through which BAR3-induced NO production by iNOS may regulate VEGF levels. Hypoxia leads to the stabilization of HIF-1α that, migrating into the nucleus, dimerizes with HIF-1β, thus triggering the transcription of a plethora of HIF-1 target genes, including those of BAR3, iNOS and VEGF. BAR3 activity (stimulated by agonists or typical BAR3 constitutive activity [88]), through a presently undetected mechanism, participates in iNOS gene expression and iNOS production of NO. The literature data report several mechanisms through which NO may impact VEGF production, both directly and indirectly. One of the direct mechanisms is based on S-nitrosylation of HIF-1α by NO, leading to the stimulation of HIF-1 transcriptional activity [99]. One of the indirect mechanisms relies on the enzyme heme oxygenase 1, which is potently induced by NO and, in turn, strongly activates VEGF gene transcription [100]. BAR3 blockade or iNOS inhibition, leading to the reduction in NO levels, would impact these direct/indirect mechanisms, downregulating VEGF production without affecting HIF-1α levels.

Together, the present results suggest that in hypoxic hRECs and MIO-M1 cells, HIF-1 activation alone seems to be insufficient to sustain VEGF production and VEGF levels are also regulated by BAR3 through its downstream effector NO. Results coming from NOS inhibition or NO donation support the existence of a functional link between BAR3 activity, NO signaling and VEGF production in hypoxic hRECs and MIO-M1 cells. Both NOS enzymes and VEGF appear to be responsive to perturbation of BAR3 signaling only under hypoxic conditions when this receptor is overexpressed, corroborating the hypothesis of a non-tonic role for BAR3 activity. A mechanism through which BAR3, NOS (iNOS in particular) and NO may impinge on VEGF production is proposed in Figure 10. Even though a correlation of these in vitro findings to the in vivo situation is difficult, they represent a first step in evaluating the functional role of the BAR3/NOS/VEGF axis in the hypoxic retina and suggest the value of this axis as a possible target in those retinal conditions in which hypoxia-induced VEGF upregulation triggers the development of neovascularization.

## Figures and Tables

**Table 1:** Forward (Fw) and reverse (Rv) primers used in qRT-PCR experiments.

Primer	Sequences
*BAR1*	Fw: 5′-GAGTGGCTTGCTGATGTTCCT-3′ Rv: 5′-AATGCTTCTCCCTTCCCCTAA-3′
*BAR2*	Fw: 5′-TTGCTGGCACCCAATAGAAGC-3′ Rv: 5′-CAGACGCTCGAACTTGGCA-3′
*BAR3*	Fw: 5′-TCCAGTGGTGCCTTACATGGT-3′ Rv: 5′-AGTGGGAAGGTAGAGGTTGTGG-3′
*VEGF-A*	Fw: 5′-GAGCCTTGCCTTGCTGCTCTAC-3′ Rv: 5′-CACCAGGGTCTCGATTGGATG-3′
*β-actin*	Fw: 5′-CATGTACGTTGCTATCCAGGC-3′ Rv: 5′-CTCCTTAATGTCACGCACGAT-3′

**Table 2:** Primary and secondary antibodies used for Western blot.

Antibody	Code	Dilution	Source
Rabbit Anti-BAR1	PA5-95742	1:1000	Invitrogen (Carlsbad, CA, USA)
Rabbit Anti-BAR2	ab182136	1:1000	Abcam (Cambridge, UK)
Mouse Anti-BAR3	sc-515763	1:500	Santa Cruz Biotechnology (Dallas, TX, USA)
Rabbit Anti-HIF1α	ab2185	1:1000	Abcam
Rabbit Anti-VEGF-A	ab214424	1:1000	Abcam
Rabbit Anti-iNOS	ab178945	1:1000	Abcam
Rabbit Anti-eNOS	9572S	1:1000	Cell Signaling (Danvers, MA, USA)
Rabbit Anti-nNOS	ab3511	1:1000	Abcam
Mouse Anti-β-actin	A2228	1:2500	Sigma-Aldrich (St. Loius, MO, USA)
Goat Anti-rabbit	1706515	1:5000	Abcam
Rabbit Anti-mouse	A9044	1:5000	Sigma-Aldrich

## Data Availability

The original contributions presented in this study are included in the article/Supplementary Materials. Further inquiries can be directed to the corresponding author.

## Supplementary Materials

The following supporting information can be downloaded at: https://www.mdpi.com/article/10.3390/ijms26094043/s1. Ref. [101] is cited in Supplementary Materials file.

## Abbreviations

The following abbreviations are used in this manuscript:

1400W	N-[[3-(aminomethyl)phenyl]methyl]-ethanimidamide, dihydrochloride
BAR	Β-adrenoceptor
DMSO	Dimethyl sulfoxide
FBS	Fetal bovine serum
HIF	Hypoxia-inducible factor
hRECs	Human retinal endothelial cells
L-NAME	Nω-nitro-L-arginine methyl ester hydrochloride
MIO-M1	Human Müller cells
MTT	3-[4,5-dimethylthiazol-2-yl]-2,5-diphenyl tetrazolium bromide
NO	Nitric oxide
eNOS	Endothelial nitric oxide synthase
iNOS	Inducible nitric oxide synthase
nNOS	Neuronal nitric oxide synthase
OD	Optical density
OIR	Oxygen-induced retinopathy
PBS	Phosphate-buffered saline
qRT-PCR	Quantitative real-time polymerase chain reaction
SNAP	S-Nitroso-N-acetyl-DL-penicillamine
VEGF	Vascular endothelial growth factor
