# Supplementary material for: Hypoxia-Dependent Upregulation of VEGF Relies on β3-Adrenoceptor Signaling in Human Retinal Endothelial and Müller Cells
Source: Int J Mol Sci. 2025 Apr 24;26(9):4043. doi: 10.3390/ijms26094043 (PMC12071845; doi:10.3390/ijms26094043)
Supplement: Supplementary file 1 [file ijms-26-04043-s001.zip › ijms-3549021-supplementary.pdf]

Supplementary Materials and Methods

Wound healing assay

hRECs were seeded in 24-well culture dishes. The seeding density was 2.4×10^5 cells per well. After cells were confluent, scratches were made with a 200 µl sterile pipette tip, then cellular debris was removed by gentle washing in PBS. Thereafter, the medium was replaced by DMEM containing 0.1% FBS in the presence or absence of either 10 µM SR59230A or DMSO and the cells were allowed to migrate for 24 h. The photos of the distance between the wound edges were taken from the same regions under a Nikon Eclipse TE200 inverted optical microscope at 0 and 24 h. The gap size was measured using Image-J 1.50 software. Data are expressed as % wound closure calculated as [premigration length (at 0h) – migration length (at 24h)]/premigration length (at 0h) x 100 as in Mazumder et al. (2016). In order to verify that a different cell number did not contribute to differential wound healing**,** after migration assay cells were trypan blue–stained and counted.

Mazumder, A.; Dwivedi, A.; du Preez, J.L.; du Plessis, J. In vitro wound healing and cytotoxic effects of sinigrin-phytosome complex. *Int. J. Pharm*. **2016,** *498*, 283-293. doi: 10.1016/j.ijpharm.2015.12.027.

BAR Silencing to Validate BAR Antibodies

Predesigned Flexitube siRNAs directed to BAR1, BAR2 or BAR3 and non-silencing control (scramble) siRNAs (Qiagen, Hilden, Germany) were used to reduce BAR expression. MIO-M1 cells were transfected with four different siRNAs *per* target according to manufacturer's instructions, using the HiPerfect Transfection Reagent (Qiagen). Cells were cultured for 24h in normoxia at 37 °C. After evaluating siRNA efficiency using qRT-PCR, the two siRNAs giving the maximum reduction of target levels were selected and used to validate commercially available antibodies targeting BAR1, BAR2 or BAR3 in Western blot experiments.

Supplementary Results

DMSO Has No Effect on the Viability of hRECs and MIO-M1 Cells

SR59230A was dissolved in DMSO and then diluted in the cell culture medium (EBM-2 for hRECs and DMEM for MIO-M1 cells) to reach the final concentration. To evaluate whether DMSO could influence cell viability we used the MTT assay. In both hRECs and MIO-M1 cells we found no difference in cell viability at the different percentages of DMSO used, neither in normoxia nor in hypoxia (Supplementary Figure S1).

Effect of SR59230A on hREC migration

To evaluate whether SR59230A may affect the angiogenic potential of hRECs, a wound healing assay was performed. As shown in Supplementary Figure S2, the scratch progressively declined over time and in both normoxic and hypoxic DMSO-treated hRECs wound healing reached almost 100% after 24 h. In normoxia, wound healing was not affected by treatment with SR59230A that, on the contrary, reduced the healing process by about 50% in hypoxia. This

was likely due to a reduced migration capability of hRECs, since after 24 h the cell number was quite the same in the different experimental conditions.

Validation of Antibodies Against BAR Subtypes

In order to validate BAR1, BAR2 and BAR3 antibodies, we silenced BAR1, BAR2 or BAR3 expression in MIO-M1 cells. We chose two of the most effective siRNAs based on qRT-PCR results (Supplementary Figure S4A–C). We then performed Western Blot experiments on cells silenced with those effective siRNAs to test the antibody specificity. The expression of BAR1, BAR2 and BAR3 was significantly reduced in silenced cells compared to controls (Supplementary Figure S4D–F), indicating specific binding of each antibody to its target protein.

SUPPLEMENTARY FIGURES

Supplementary Figure S1

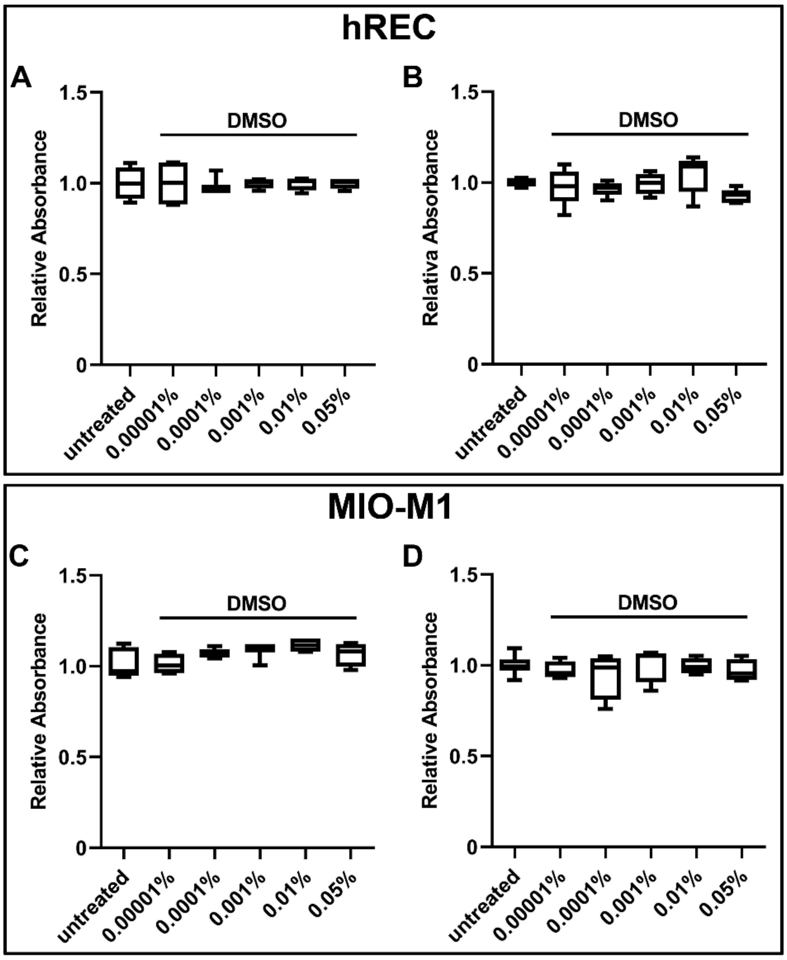

Supplementary Figure S1. Effect of DMSO on hREC and MIO-M1 cell proliferation. Cell proliferation of hRECs (**A,B**) and MIO-M1 cells (**C,D**) was analyzed through MTT assay in response to the indicated concentrations of DMSO, both in normoxia (**A,C**) and in hypoxia (**B,D**). Data are shown as box plots with minimum to maximum whiskers and represent the absorbance values normalized to those measured in untreated controls (n = 4). Statistical significance was evaluated through one-way ANOVA followed by Tukey's post-hoc multiple comparison test.

Supplementary Figure S2

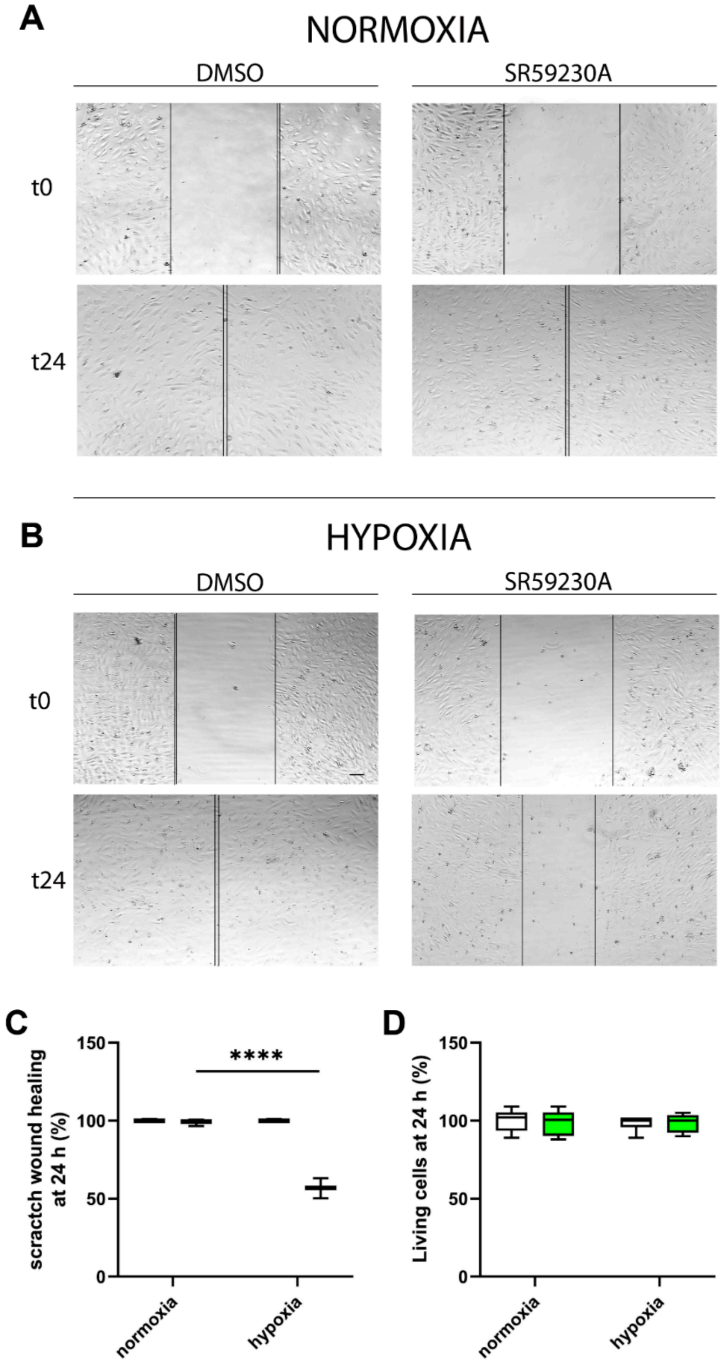

Supplementary Figure S2. Effect of 10 μM SR59230A on hREC migration. Cell migration was evaluated using a wound healing assay. (**A,B**) Scratch wound healing closure at t0 and after 24 h after treatment with DMSO or SR59230A in normoxic (**A**) or hypoxic (**B**) conditions. (**C**) Evaluation of scratch wound healing progression reported as percentage values. (**D**) Number of living cells calculated after 24 h in the different experimental conditions using the trypan blue assay. In each data set of C and D, the first box corresponds to DMSO treatment, while the second box corresponds to SR59230A treatment. Data are shown as box plots with minimum to maximum whiskers (n = 5). Statistical significance was evaluated through one-way ANOVA followed by Tukey's post-hoc multiple comparison test.

Supplementary Figure S3

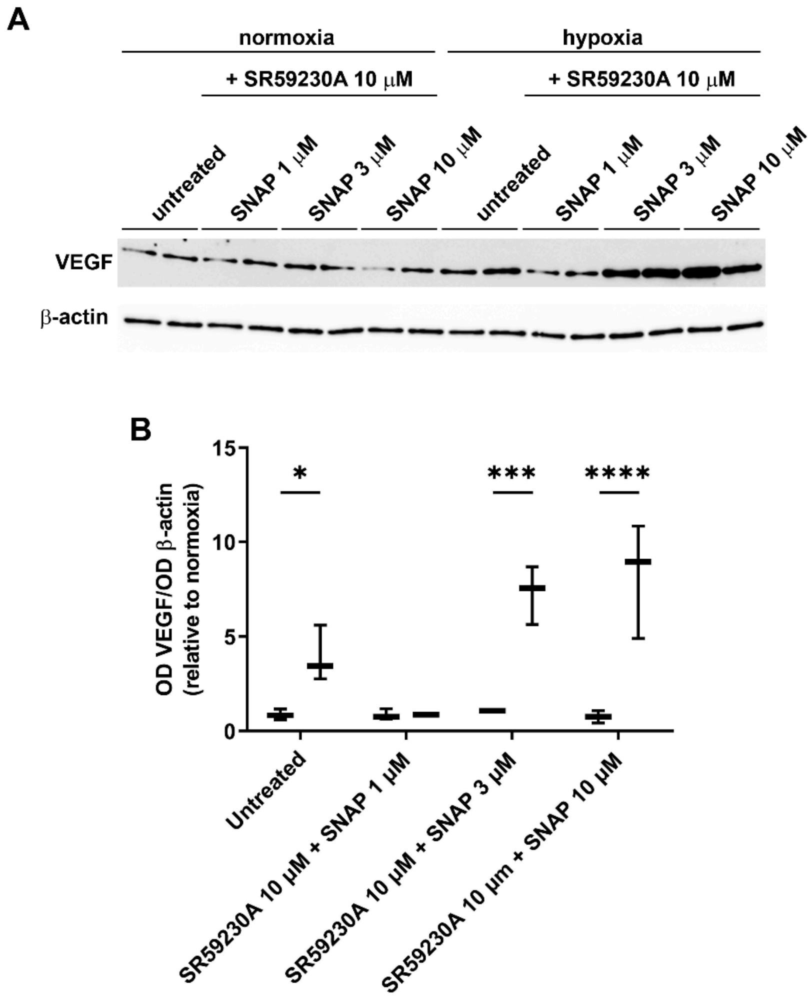

Supplementary Figure S3. Effect of 10 μM SR59230A in the presence of different concentrations of the NO donor SNAP on the expression of VEGF in MIO-M1 cells exposed to normoxia or 24 h hypoxia. (**A, B**) Representative Western blot (**A**) and densitometric analysis (**B**) of VEGF levels. MIO-M1 cells were cultured in normoxia (first box in each data set) or hypoxia (second box in each data set). β-actin was used as the loading control and OD values were normalized to those measured in untreated normoxic controls (n = 3). Data are shown as box plots with minimum to maximum whiskers. Statistical significance was evaluated through two-way ANOVA followed by Bonferroni's multiple comparisons post-hoc test. *$p < 0.05$, *** $p < 0.001$ and **** $p < 0.0001$.

Supplementary Figure S4

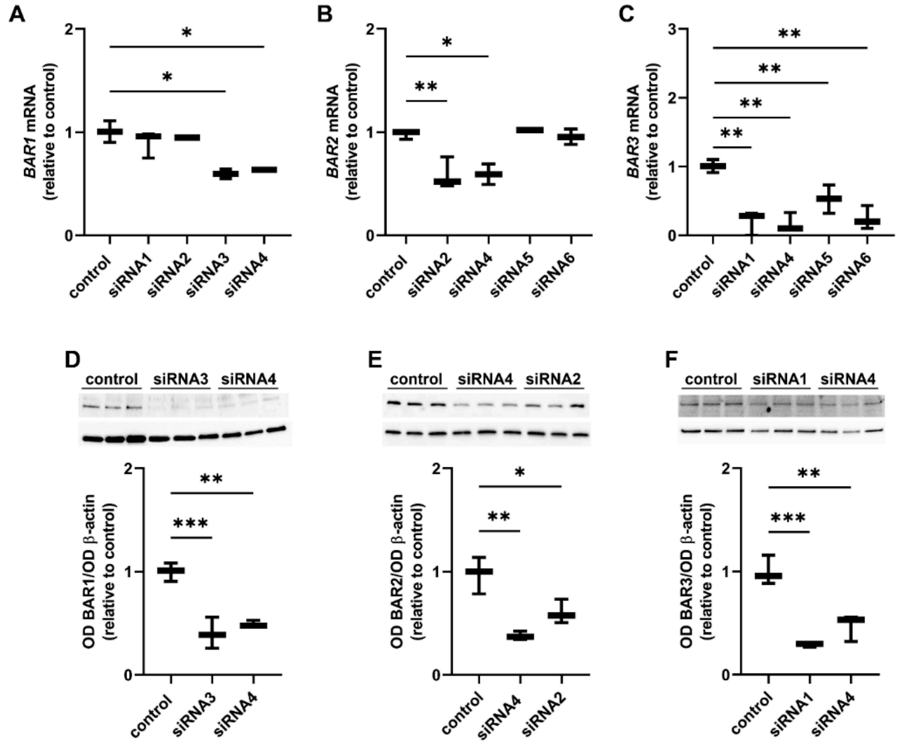

Supplementary Figure S4. Validation of BAR antibodies through siRNA silencing of BAR expression in MIO-M1 cells. (**A-C**) mRNA levels of *BAR1* (**A**), *BAR2* (**B**) and *BAR3* (**C**) after silencing with four different siRNAs for each target. (**D-F**) Representative Western blots and densitometric analysis of BAR1 (**D**), BAR2 (**E**) and BAR3 (**F**) after silencing with the two most effective siRNAs identified in (**A-C**). In (**A-C**) data were analyzed by the formula $2^{-\Delta\Delta}$CT using β-actin as the internal standard and normalized to those measured in controls (n = 3). In (**D-F**) β-actin was used as the loading control and OD values were normalized to those measured in controls (non-silencing siRNAs; n = 3). Data are shown as box plots with minimum to maximum whiskers. Statistical significance was evaluated through one-way ANOVA followed by Tukey's post-hoc multiple comparison test. *$p < 0.05$, **$p < 0.01$ and ***$p < 0.001$.
